# Long-term efficacy of lipoprotein apheresis and lomitapide in the treatment of homozygous familial hypercholesterolemia (HoFH): a cross-national retrospective survey

**DOI:** 10.1186/s13023-021-01999-8

**Published:** 2021-09-08

**Authors:** Laura D’Erasmo, Antonio Gallo, Angelo Baldassare Cefalù, Alessia Di Costanzo, Samir Saheb, Antonina Giammanco, Maurizio Averna, Alessio Buonaiuto, Gabriella Iannuzzo, Giuliana Fortunato, Arturo Puja, Tiziana Montalcini, Chiara Pavanello, Laura Calabresi, Giovanni Battista Vigna, Marco Bucci, Katia Bonomo, Fabio Nota, Tiziana Sampietro, Francesco Sbrana, Patrizia Suppressa, Carlo Sabbà, Fabio Fimiani, Arturo Cesaro, Paolo Calabrò, Silvia Palmisano, Sergio D’Addato, Livia Pisciotta, Stefano Bertolini, Randa Bittar, Olga Kalmykova, Sophie Béliard, Alain Carrié, Marcello Arca, Eric Bruckert

**Affiliations:** 1 Department of Translational and Precision Medicine, Sapienza University of Rome, Viale del Policlinico 155, Rome, Italy; 2 Department of Endocrinology and Cardiovascular Disease Prevention, Assistance Publique-Hôpitaux de Paris, La Pitié-Salpêtrière Hospital, Sorbonne University, Paris, France; 3 Sorbonne Université, UPMC Univ Paris 06, INSERM 1146, - CNRS 7371, Laboratoire d’imagerie Biomédicale, Paris, France; 4 Dipartimento di Promozione Della Salute, Materno Infantile, Medicina Interna e Specialistica di Eccellenza “G. D’Alessandro” (PROMISE), Università Degli Studi Di Palermo, Palermo, Italy; 5 Department of Clinical Medicine and Surgery, Federico II University, Naples, Italy; 6 Department of Molecular Medicine and Medical Biotechnology, University of Naples Federico II, Naples, Italy; 7 CEINGE, Advanced Biotechnology, Naples, Italy; 8 Department of Medical and Surgical Sciences, University Magna Graecia, Catanzaro, Italy; 9 Centro Grossi Paoletti, Dipartimento di Scienze Farmacologiche e Biomolecolari, Università Degli Studi di Milano, Milan, Italy; 10 Medical Department, Azienda Ospedaliero-Universitaria di Ferrara, Ferrara, Italy; 11 Dipartimento di Medicina e Scienze Dell’Invecchiamento, Università Degli Studi “G. d’annunzio” di Chieti, Pescara, Italy; 12 Metabolic Disease and Diabetes Unit, AOU San Luigi Gonzaga, Orbassano’, Turin, Italy; 13 Lipoapheresis Unit-Reference Center for Diagnosis and Treatment of Inherited Dyslipidemias, Fondazione Toscana “Gabriele Monasterio”, Via Moruzzi 1, Pisa, Italy; 14 Department of Internal Medicine and Rare Disease Centre “C.Frugoni”, University Hospital of Bari “A. Moro”, Piazza G. Cesare 11, Bari, Italy; 15 Division of Clinical Cardiology, Department of Translational Medical Sciences, University of Campania “Luigi Vanvitelli”, A.O.R.N. Sant’ Anna e San Sebastiano, 81100 Caserta, Italy; 16 Hypertension and Atherosclerosis Research Group, Medical and Surgical Sciences Department, Sant’Orsola-Malpighi University Hospital, Via Albertoni 15, 40138 Bologna, Italy; 17 Department of Internal Medicine, University of Genoa, Genoa, Italy; 18 IRCCS-Polyclinic Hospital San Martino, Genoa, Italy; 19 Inserm, Institute of Cardiometabolism and Nutrition (ICAN), UMR_S1166, Department of Metabolic Biochemistry, Assistance Publique, Hôpitaux de Paris, Hôpital de La Pitié-Salpêtrière, Sorbonne University, Paris, France; 20 Aix Marseille University, INSERM, INRA, C2VN, Marseille, France; 21 Department of Nutrition, Metabolic Diseases, Endocrinology, La Conception Hospital, Marseille, France; 22 Inserm, Institute of Cardiometabolism and Nutrition (ICAN), UMR_S1166, APHP, Department of Biochemistry, Obesity and Dyslipidemia Genetics Unit, Hôpital de La Pitié, Sorbonne University, Paris, France

**Keywords:** Homozygous hypercholesterolemia, Lipoprotein apheresis, Lomitapide, LDL, Therapeutics, Genetic disease, Cholesterol burden

## Abstract

**Background:**

Homozygous familial hypercholesterolemia (HoFH) is a rare life-threatening condition that represents a therapeutic challenge. The vast majority of HoFH patients fail to achieve LDL-C targets when treated with the standard protocol, which associates maximally tolerated dose of lipid-lowering medications with lipoprotein apheresis (LA). Lomitapide is an emerging therapy in HoFH, but its place in the treatment algorithm is disputed because a comparison of its long-term efficacy versus LA in reducing LDL-C burden is not available. We assessed changes in long-term LDL-C burden and goals achievement in two independent HoFH patients’ cohorts, one treated with lomitapide in Italy (n = 30) and the other with LA in France (n = 29).

**Results:**

The two cohorts differed significantly for genotype (p = 0.004), baseline lipid profile (p < 0.001), age of treatment initiation (p < 0.001), occurrence of cardiovascular disease (p = 0.003) as well as follow-up duration (p < 0.001). The adjunct of lomitapide to conventional lipid-lowering therapies determined an additional 58.0% reduction of last visit LDL-C levels, compared to 37.1% when LA was added (*p*_adj_ = 0.004). Yearly on-treatment LDL-C < 70 mg/dl and < 55 mg/dl goals were only achieved in 45.5% and 13.5% of HoFH patients treated with lomitapide. The long-term exposure to LDL-C burden was found to be higher in LA than in Lomitapide cohort (13,236.1 ± 5492.1 vs. 11,656.6 ± 4730.9 mg/dL-year respectively, *p*_adj_ = 0.002). A trend towards fewer total cardiovascular events was observed in the Lomitapide than in the LA cohort.

**Conclusions:**

In comparison with LA**,** lomitapide appears to provide a better control of LDL-C in HoFH. Further studies are needed to confirm this data and establish whether this translates into a reduction of cardiovascular risk.

**Supplementary Information:**

The online version contains supplementary material available at 10.1186/s13023-021-01999-8.

## Background

Homozygous familial hypercholesterolemia (HoFH) is a rare inherited disorder of lipid metabolism characterized by marked elevation of low-density lipoprotein cholesterol (LDL-C) caused by an almost abolished function of LDL receptor (LDLR) [1]. Typically, HoFH patients develop clinical complications of atherosclerotic cardiovascular disease (ASCVD) early in life [2–5] due to the very high life-long LDL-C burden associated with this condition [6, 7]. Therefore, an early, intensive LDL-C lowering therapy is mandatory to control the high risk of ASCVD in HoFH.

Lipoprotein apheresis (LA) in addition to statins and ezetimibe is considered the standard of therapy in HoFH [1, 8]. LA transiently removes LDL particles from plasma during extracorporeal circulation by selectively binding apolipoprotein B (ApoB)-containing lipoproteins and has been demonstrated to be very effective in lowering LDL-C levels [8, 9]. However, an optimal inter-procedures LDL-C control is difficult to reach due to the post-treatment LDL-C rebound and, therefore, the residual cardiovascular risk of these patients remains high [10].

In recent years, promising new drugs in the treatment of HoFH have become available [11]. Monoclonal antibodies inhibiting proprotein convertase subtilisin/kexin type 9 (PCSK9i) have been reported to decrease LDL-C by an additional 24% when added to background lipid-lowering therapies in HoFH [11]. However, PCSK9i are mostly effective in patients with residual LDLR function, showing very limited effect in those carrying null mutation or homozygous mutation in the *LDLRAP1* gene [12].

Lomitapide, an inhibitor of microsomal triglycerides transferase protein (MTP), has been proven to decrease by almost 50% LDL-C levels in HoFH in adjunct to other lipid-lowering medications (including LA) [13]. Recent real-world studies have confirmed the efficacy and safety of lomitapide in the treatment of adults and children with HoFH [11–14]. As lomitapide acts by decreasing liver secretion of VLDL and, consequently, VLDL-derived LDL production [15], its efficacy appears to be independent from the severity of functional impairment of LDLR pathway [16]. Some real-word studies have reported that the addition of lomitapide to background therapies results in the discontinuation of LA in many patients with HoFH [14].

The advent of novel therapeutic options is rapidly changing the therapeutic armamentarium for the management HoFH. Therefore, some efforts must be done to compare the effectiveness of emerging versus standard therapies in controlling LDL-C burden in these patients. Considering the rarity of the disease and the fact that large and formal randomized clinical trials aimed at comparing lomitapide versus LA are not feasible, we believe that a real-world survey might help in clarifying the benefit of these two treatments. However, a comparison of benefit of lomitapide versus LA in the long-term control of LDL-C burden in HoFH is not available to date.

Our aim was to indirectly compare the long-term LDL-lowering effectiveness of these two treatments throughout the evaluation of two independent HoFH cohorts treated with lomitapide or LA. We therefore assessed LDL-C reduction, target achievement and long-term LDL-C burden in these two populations. As an exploratory analysis, we also aimed at evaluating major atherosclerotic cardiovascular events (MACE) incidence during follow-up in the two cohorts.

## Results

The two cohorts differed significant for demographic, clinical, genetic, and biochemical characteristics as shown in Table 1. The list of causative HoFH mutations is reported in the Additional file 1: Table 1.

**Table 1:** Baseline clinical characteristics of HoFH patients

	Lomitapide cohort (N = 30)	LA cohort (N = 29)	*p*
Geographic origin			
European Non-Finnish-Southern European, n (%)	29 (96.7)	10 (34.5)	< 0.001
Others, n (%)^a^	1 (3.3)	19 (65.5)	
Demographic			
Age, years	40.0 (27.5–56.5)	18.0 (10.5–30.5)	< 0.001
Male, n (%)	15 (50.0)	14.0 (48.3)	NS
Genotype			
Uncertain^b^	12 (40.0)	13 (44.8)	0.004
ARH	6 (20.0)	2 (6.9)	
Defective/defective	9 (30.0)	1 (3.4)	
Null/defective	1 (3.3)	1 (3.4)	
Null/null	2 (6.7)	12 (41.4)	
Xanthomata, n (%)	26 (86.7)	27 (93.1)	NS
Risk factors, n (%)			
Smoking	4 (13.3)	2 (6.9)	NS
T2DM	1 (3.3)	0	NS
HTN	11 (36.7)	0	< 0.001
Plasma lipids (mg/dl)			
Untreated LDL-C^c^	481.4 ± 153.1	794.3 ± 344.2	0.01
Lowest LDL-C on conventional LLT before LA/Lomitapide	246.5 (170.3–295.8)	502.0 (309.5–606.0)	< 0.001
Pre-treatment LDL-C burden (mg/dL-year)	11,463.9 (6751.5–14,468.9)	7313.5 (4302.1–11,451.3)	0.034
Baseline TC	357.6 ± 136.5	510.1 ± 183.6	0.001
Baseline HDL-C	44.7 ± 12.9	30.3 ± 9.5	< 0.001
Baseline LDL-C	272.5 ± 108.8	453.0 ± 179.5	< 0.001
Baseline TG	96.5 (66.8–132.0)	82.5 (59.8–144.0)	NS
LLT, n (%)			
None	1 (3.3)	0	NS
LA	8 (26.7)	0^d^	0.003
PCKS9i	6 (20.0)	0	0.011
Statin	29 (96.7)	28 (96.6)	NS
Ezetimibe	27 (90.0)	11 (37.9)	< 0.001
Fibrate	1 (3.3)	2 (6.9)	NS
Porto-caval shunt	0	2 (6.9)	NS
Resins	0	9 (31.0)	0.001
Major atherosclerotic cardiovascular events (MACE)			
Age at first MACE	35.0 (30.0–52.5)	19.0 (14.0–35.0)	0.003
Total MACE, n (%)	17 (56.7)	13 (44.8)	NS
CHD	15 (50.0)	9 (31.0)	
Stroke	0	1 (3.4)	
PAD	1 (3.3)	3 (10.3)	
Carotid revascularization	5 (16.7)	2 (6.9)	
Aortic valve replacement	4 (14.3)	5 (19.2)	

Data are represented median (interquartile range) and number (percentage) as appropriate. Pre-treatment LDL-C burden was calculated as: $\left({LDL-C}_{baseline}*{ Age }_{first LA or Lomitapide prescrption}\right)$

ARH, Autosomal Recessive Homozygous; T2DM, type 2 diabetes; HTN, hypertension; LLT, Lipid Lowering Therapy; LDL-C, low density lipoprotein cholesterol; TC, total cholesterol; HDL-C, high density lipoprotein cholesterol; TG, triglycerides; BMI, body mass index; HTN, hypertension; T2DM, type 2 diabetes; LA, Liporprotein apheresis; PCKS9i, Proprotein convertase subtilisin/kexin type 9 inhibitors; MACE, Major Atherosclerotic Cardiovascular Events; CHD, coronary heart disease; PAD, peripheral artery disease; NS, not significant

^a^1 patient from Africa, 9 from North-Africa, 1 from central-eastern Europe, 1 from Antille, 3 from South Asia and 3 from Turkey (for one patient the information on ethnicity was not available)

^b^The genotype was defined as uncertain if molecular diagnosis was not available or the molecular testing indicated the presence of variants of uncertain significance (VUS)

^c^Untreated LDL-C values were available for 21 and 12 subjects in the Lomitapide and LA cohort, respectively

^d^LA was started at this visit

Patients from the LA cohort showed a more severe genotype as compared to the Lomitapide cohort (*p* = 0.004). The age of initiation of treatment with LA was earlier than that of lomitapide (*p* < 0.001). First ASCVD occurred earlier in the LA cohort (*p* = 0.003), while the overall prevalence of ASCVD did not differ between the two cohorts.

Patients in the Lomitapide cohort exhibited lower values of untreated (*p* < 0.001), lowest on conventional lipid-lowering treatment (LLT) (*p* = 0.01) or baseline (*p* < 0.001) LDL-C as compared with those in the LA cohort.

### Lipid changes during follow-up

The median follow-up duration was 16.7 years in the LA cohort and 2.3 years in Lomitapide cohort (*p* < 0.001) (Table 2).

**Table 2:** Follow-up lipid profile, lipid lowering therapies and MACE in HoFH cohorts

	Lomitapide cohort (N = 30)	LA cohort (N = 29)	*p*
Duration of follow-up (years)	2.2 (1.0–3.7)	16.5 (9.0–25.5)	< 0.001
Risk factors, n (%)			
Smoking	2 (6.7)	7 (24.1)	0.006
T2DM	1 (3.3)	0	NS
HTN	11 (36.7)	7 (24.1)	NS
Plasma lipids (mg/dl)			
On-treatment LDL-C	111.4 (83.7–177.8)	247.2 (217.9–340.8)	< 0.001
On-treatment LDL-C burden (mg/dL-year)	293.0 (153.0–454.8)	3849.3 (2238.3–7045.5)	< 0.001
Last visit TC	169.0 (126.2 – 276.7)	390.5 (321.9 – 500.0)	< 0.001
Last visit HDL-C	47.5 (38.7 – 60.0)	30.6 (19.7 – 40.8)	0.001
Last visit LDL-C	114.0 (64.7 – 202.95)	340.5 (280.5 – 418.8)	< 0.001
Last visit TG	58.0 (35.0 – 81.0)	92.7 (65.7 – 152.9)	0.001
LLT at last visit, n (%)			
LA	0	29 (100)	< 0.001
PCKS9i	5 (16.7)	1 (3.4)	NS
Statin	29 (96.7)	27 (93.1)	NS
Ezetimibe	28 (93.3)	27 (93.1)	NS
Fibrate	0	0	-
Porto-caval shunt	0	1 (3.4)	NS
Resins	0	0	-
Incident MACE			
Cumulative number of MACE	7	42	-
Individual MACE event, n (%)	4 (13.3)	16 (55.2)	0.001
CHD	3 (10.0)	11 (37.9)	0.012
Stroke	0	2 (6.9)	NS
PAD	0	14 (48.3)	0.001
Carotid revascularization	1 (3.3)	2 (6.9)	NS
Aortic Valve replacement	1 (3.3)	6 (22.2)	0.048
Death for cardiovascular disease	1 (3.3)	4 (14.3)	NS
Death for other reason	0	1 (3.6)	NS
Number of MACE per patient, n (%)			
			0.003
0	26 (86.7)	13 (44.8)	
1	3 (6.7)	6 (20.7)	
≥ 2	2 (6.7)	10 (34.5)	
IR *per* 1000 person/years	77.6	86.9	-

Data are represented as median (interquartile range), mean (standard deviation) or number (percentage) as appropriate. For estimation of incident MACE and Incident Rate of incident MACE (× 1000 person-years) please refer to Methods. Duration of follow-up has been calculated as the difference between the date of the last and the baseline visit with LA or lomitapide. On-treatment LDL-C burden was calculated as: $({LDL-C}_{follow-up}*{Years}_{follow-up})$

T2DM, type 2 diabetes; HTN, hypertension; LDL-C, low density lipoprotein cholesterol; TC, total cholesterol; HDL-C, high density lipoprotein cholesterol; TG, triglycerides; LLT, Lipid Lowering Therapy; LA, Lipoprotein apheresis; PCKS9i, Proprotein convertase subtilisin/kexin type 9 inhibitors; MACE, Major Atherosclerotic Cardiovascular Events; CHD, coronary heart disease; PAD, peripheral artery disease; IR, incident rate; NS, not significant

**Fig. 1:**
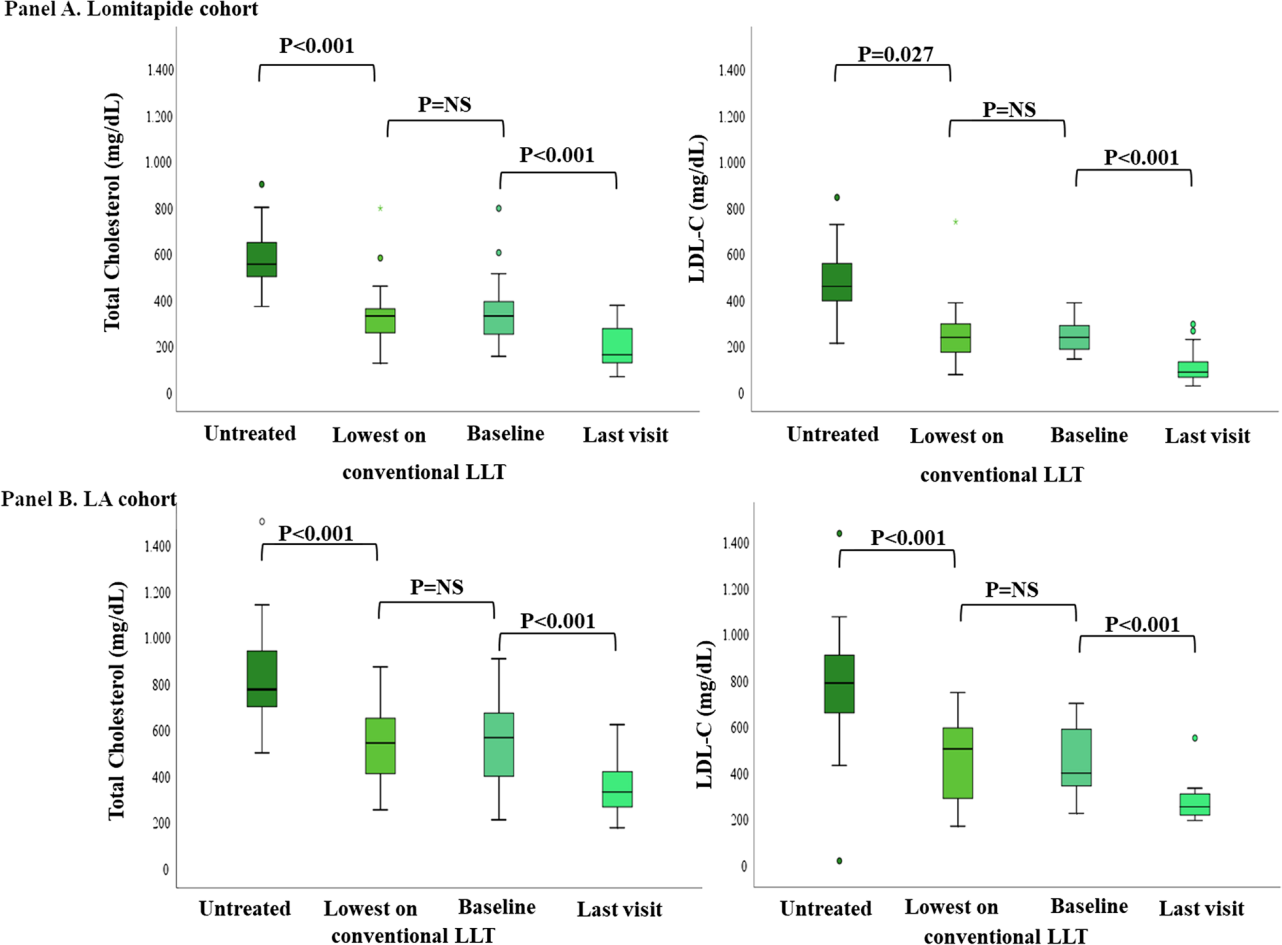
Total and LDL cholesterol changes in HoFH according to lipid-lowering therapies. Box plot graphs represent median values of TC and LDL-Cs. Lipid values are shown in 4 different shades of green corresponding to the untreated, lowest on conventional LLT, baseline (before initiation of lomitapide or LA treatment) and at last visit measurements. For the definition of untreated, lowest values on conventional LLT, baseline and last visit see Material and Methods. In **A** are reported data observed in the lomitapide cohort whereas in **B** those in the LA cohort. LDL-C, low density lipoprotein cholesterol; LLT, lipid-lowering therapy; LA, lipoprotein apheresis; NS, not significant

Figure 1 shows changes in TC and LDL-C concentrations in the two cohorts before and after addition of lomitapide or LA. The addition of lomitapide to standard LLT resulted in a further 58.0% reduction in the last visit LDL-C, compared to 37.1% LDL-C reduction after the addition of LA (*p* = 0.013). These results were independent from genotype, ethnicity, untreated LDL-C levels, gender and age at baseline (Lomitapide vs. LA cohort β -0.673 *p*_adj_ = 0.004, data not shown).

**Fig. 2:**
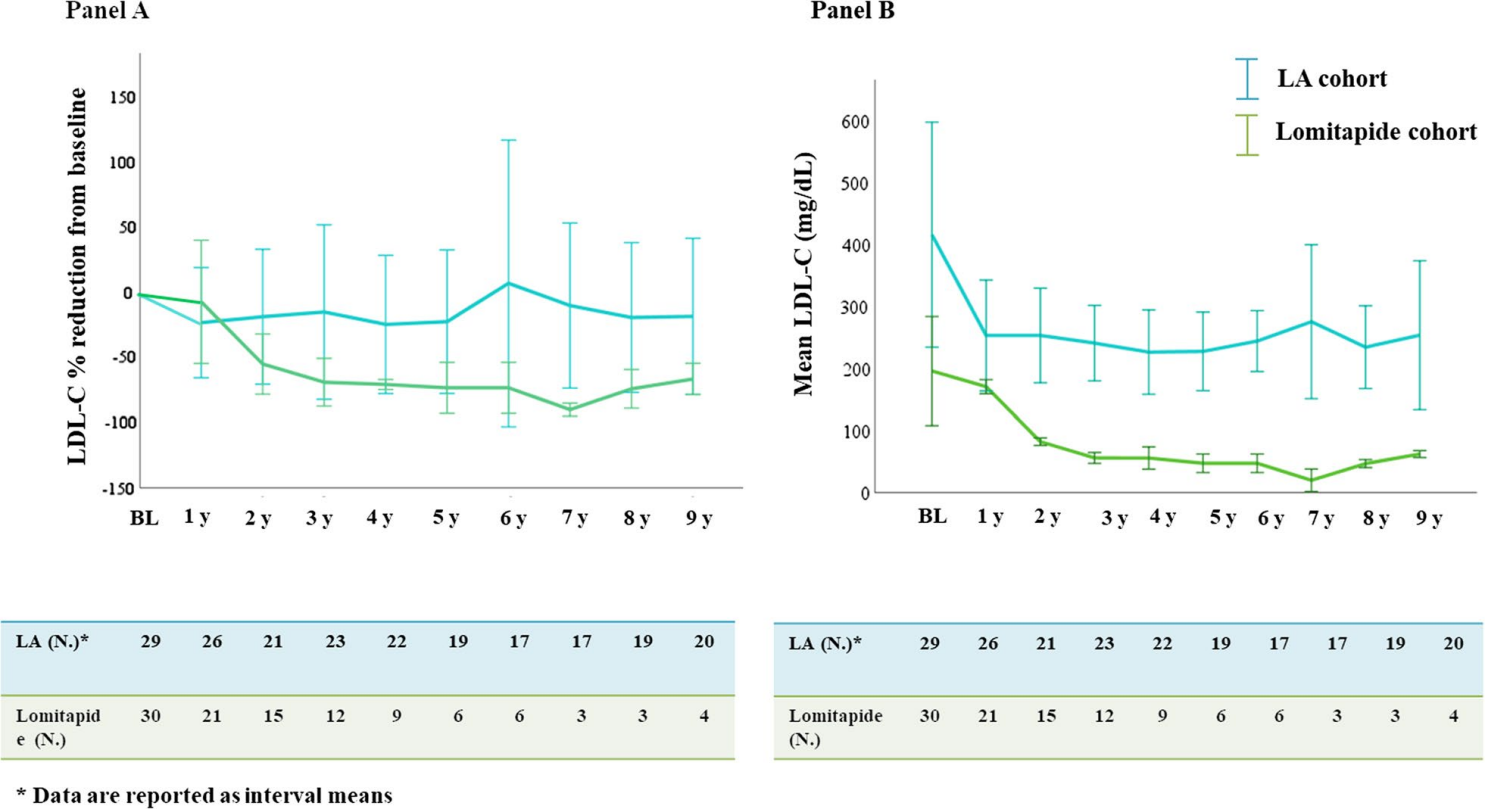
Yearly changes of LDL-C in Lomitapide (green) and LA (light blue) cohort during follow-up. As the maximum duration of follow-up in the Lomitapide cohort was about 9 years, we compared the two cohorts in the same interval of time. Data are reported as mean values ± 2 standard errors per each time-point. **A.** HoFH subjects treated with lomitapide achieved significantly greater mean yearly LDL-C percent reduction from baseline as compared with those on LA. Data are reported as mean percent reduction ± 2 standard errors per each time-point. **B.** HoFH subjects treated with lomitapide achieved significantly lower mean yearly LDL-C values as compared with those on LA. P_adj,_ value is adjusted for genotype, ethnicity, gender, untreated LDL-C and age at baseline. BL, baseline; LDL-C, low density lipoprotein cholesterol; LA, lipoprotein apheresis

**Fig. 3:**
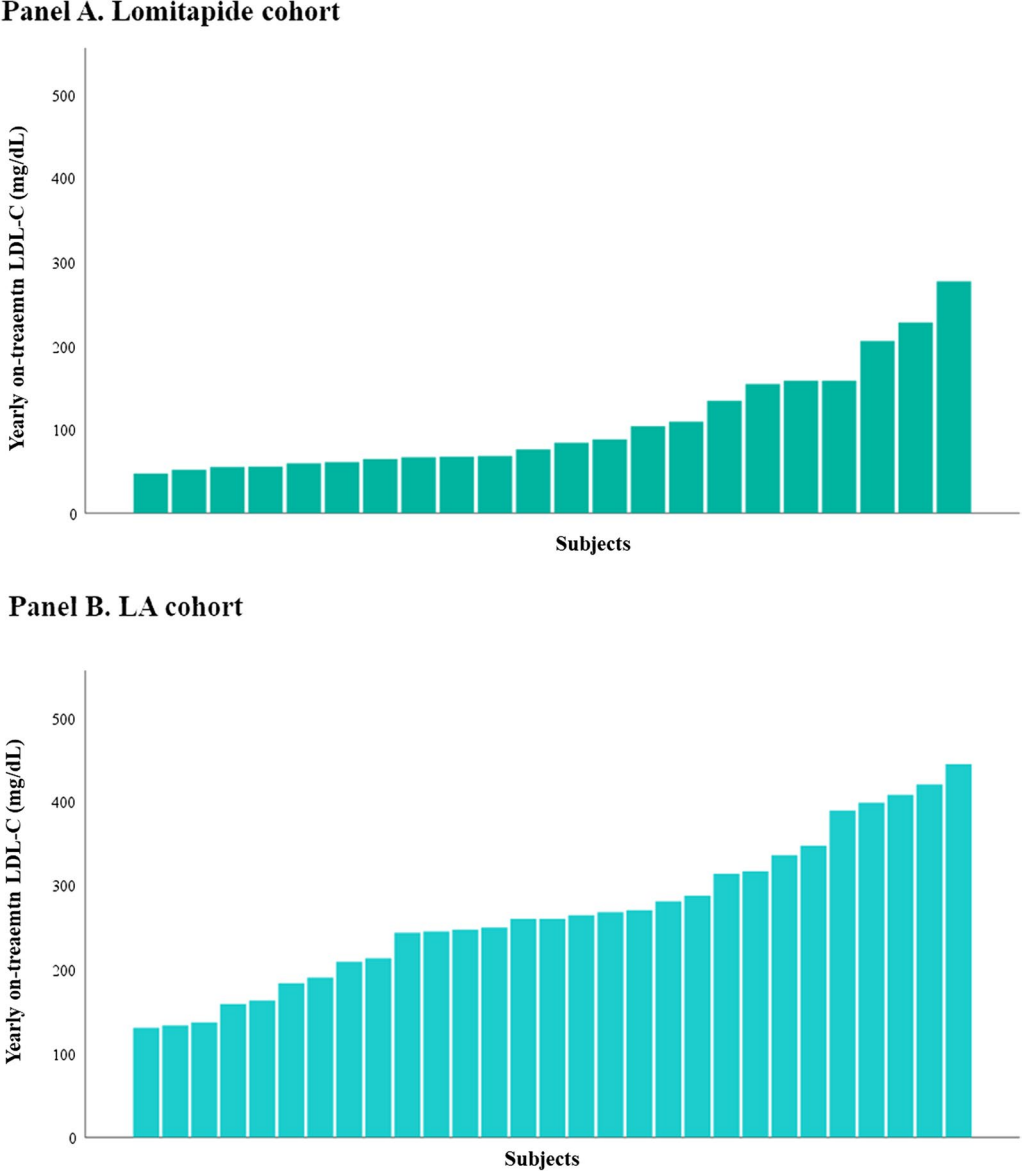
Individual yearly on-treatment LDL-C levels in Lomitapide and LA cohort. Individual yearly on-treatment LDL-C has been calculated as the average of yearly LDL-C measurements. In **A** are reported data in the Lomitapide cohort whereas in **B** those in the LA cohort. LDL-C, low density lipoprotein cholesterol; LA, Lipoprotein apheresis

The yearly variation of LDL-C values in the two cohorts is reported in Fig. 2. The mean yearly on-treatment LDL-C percent reduction (Panel A) as well as the absolute yearly on-treatment LDL-C levels (Panel B) at 9 years were significantly lower in the Lomitapide cohort as compared with the LA cohort and this difference persisted after adjusting for genotype, gender, untreated LDL-C, age at baseline and ethnicity (respectively *p*_*adj*_ < 0.001 for both). The percentage of subjects that achieved a yearly on-treatment LDL-C percent reduction greater that 50% from baseline was 77.3% (N = 17) in the Lomitapide cohort and 24.1% (N = 7) in the LA cohort (*p* < 0.001). Within the Lomitapide cohort, 45.5% and 13.6% HoFH patients reached a yearly on-treatment LDL-C value of LDL-C ≤ 70 mg/dl or 55 mg/dl, respectively [Pearson chi-squared = 16.4, *p* = 0.001 vs. LA cohort, Fig. 3 (Panel A) and Pearson chi-squared 4.2, *p* = 0.04 vs. LA cohort, Fig. 3 (Panel B)].

Finally, when we estimated the LDL-C burden, HoFH patients exposed to lomitapide exhibited a significantly lower total LDL-C burden as compared with patients in the LA cohort and this persisted after adjustment for age at follow-up, untreated LDL-C values, genotype, ethnicity, gender (11,656.6 ± 4730.9 mg/dL-year vs*.* 13,236.1 ± 5492.1 mg/dL-year, *p*_*adj*_ = 0.002, Additional file 2: Fig. 1, Panel A). Indeed, as compared to values before adding lomitapide, LDL-C burden at last visit decreased by 97.9% (Additional file 2: Fig. 1, Panel B), while after addition of LA this parameter was reduced by 52.0% (Additional file 2: Fig. 1, Panel C). These results were confirmed after adjustment for untreated LDL-C values, genotype, ethnicity, gender (*p*_*adj*_ = 0.045) and were not related to the ongoing background LLT (Table 2).

### Cardiovascular outcomes

As an exploratory analysis, we compared the occurrence of MACE during follow-up in HoFH patients receiving lomitapide or LA. In this analysis we included only patients that had been on treatment for at least 1 year. The absolute number of incident MACE in the Lomitapide cohort was lower than that in the LA cohort (13.3% vs. 55.2%; *p* < 0.001). When we estimated the MACE IR standardized for time of exposure, the IR in the Lomitapide was lower than that in the LA cohort (77.6 vs. 86.9 per 1000 person/years) (Table 2). Results of the multivariate Cox regression analysis did not reveal significant difference in the risk of incident MACE (or its recurrence) in Lomitapide as compared with LA cohort.

## Discussion

Our findings suggest that lomitapide in addition to statin/ezetimibe treatment might be more efficacious than LA in the long-term reduction of LDL-C. Indeed, HoFH patients receiving lomitapide presented larger LDL-C percent reduction in the last visit as compared to those on LA and the proportion of patients reaching a yearly on-treatment LDL-C < 70 mg/dl was higher in those treated with lomitapide than in those with LA. Same results were obtained when we analysed changes in the mean percent reduction of LDL-C during time (up to 9 years) showing that patients in the Lomitapide cohort achieved a greater LDL-C percent reduction from baseline independently from possible confounders. Finally, the reduction of cumulative LDL-C burden, which is considered a strong predictor of risk of vascular damage in FH [6, 17], was larger in patient exposed to lomitapide than in those exposed to LA.

The LDL-C goals achieved in our LA cohort are consistent with those reported in previous studies where LA was used as the sole therapeutic intervention [6, 7, 18]. Recently published data from the UK Lipoprotein Apheresis Registry including both HeFH (N = 58) and HoFH (N = 30) have shown that the overall reduction in LDL-C interval means was 43.14% [19]. Furthermore, in a previous analysis by Bruckert et al. [6], authors found a reduction in TC by 20% in LA treated patients. On the other hand, the LDL-C lowering efficacy of lomitapide was almost superimposable to that already described in Phase 3 trial [13] and real-world study [14].

As an exploratory analysis, we noted that HoFH patients who were exposed to lomitapide experienced less cardiovascular events than those who had been treated with LA and this effect was particularly evident for CHD, PAD and aortic valve replacement events. Albeit this observation must be taken with great caution due to the nature of the comparison and the size of cohorts, it may suggest a benefit of lomitapide on cardiovascular risk. One might speculate that this might be related, at least to some extent, to the increased efficacy of lomitapide in reducing the burden of LDL-C. In fact, the role of cholesterol lifelong exposure on cardiovascular risk in HoFH has been widely explored. Previous data from the French HoFH cohort showed that the cumulative total cholesterol exposure was highly associated with the incidence of MACE [6]. Accordingly, Thompson et al. [7] insisted on the role of on-treatment LDL-C levels as the main determinant of overall HoFH survival. Also, Raal et al.[18] showed that the cardiovascular protection in HoFH was related to the on-treatment LDL-C levels, estimating that a LDL-C reduction of about 30% translated into almost 50% reduction of risk. However, further studies are needed to confirm this observation definitively establishing the role of lomitapide in reducing cardiovascular risk in HoFH.

Safety data in the two cohorts have been described in detail elsewhere [14, 20]. The most common adverse events (AE) associated with LA included hypotension, nausea, abdominal discomfort, tingling, chest pain, vasovagal reaction and prolonged bleeding from anticoagulant. Anemia was another common adverse event easily managed by iron supplementation. In addition, patients on ACE-inhibitors before the beginning of LA were systematically withdrawn from the treatment in order to prevent anaphylactic-like reactions [21, 22]. Conversely, as expected the most common AE in the Lomitapide cohort was diarrhea that occurred in 41.2% of cases (data not shown). The proportion of patients referring gastrointestinal AE, however after 1 year of treatment, decreased to 16.6% after 1 year of treatment. One patient experienced liver function test elevation (> 5 ULN) after 3 months of treatment that was managed by holding lomitapide and statins and then making rechallenge (data not shown).

Some final considerations deserve the cost analysis of both LA and lomitapide treatments. Although this issue is difficult to be handled since the process of reimbursement is different in France and Italy, the direct costs of these therapies appear to be quite comparable between the two countries. Considering a weekly LA regimen treatment, the expected annual cost of LA would be about 90,000 euros while that of lomitapide is 180,000 euros. This economic evaluation does not take into account the social and personal costs of LA, as a result of the number of lost working days, not to mention the psychological burden. Further studies need to be done to better explore the pharmaco-economic aspect of these two therapies thus better allowing to compare the cost–benefit of these two treatments.

## Limitations

We acknowledge several weaknesses of the present study: the retrospective, cross-sectional observational design, and the heterogeneity of the two cohorts.

Patients from the LA cohort exhibited higher untreated and baseline LDL-C values: this observation might be explained by the higher variability in geographic origin in these patients, which can result in different socio-cultural habits having a different impact on the overall lipid profile. Nevertheless, it can be the reflection of an overall lower efficacy of baseline treatment in relation with a longer follow-up: high-dose statins were not used in HoFH children until last decade (and age at first diagnosis was lower in the LA cohort), and ezetimibe was only introduced in 2005. Other treatments, as fibrates and cholestyramine, which were used in the LA cohort primarily because of patients’ age, have a more limited effect on LDL-C than high-dose statins plus ezetimibe.

In addition, LDL-C response to treatments might have been also influenced both by the higher percentage of patients classified as carrying null/null mutations and by a more heterogeneous geographic origin within the LA cohort. However, it must be noted that in order to limit the confounding effect of these factors on the results, the efficacy of lomitapide versus LA has been evaluated adjusting for both the severity of genotype and the geographic origin.

It is important to note that we did not systematically collect safety data as this was not the aim of the present study that was primarily focused on describing the independent efficacy of these two treatments. More studies are needed to address this point in order to better clarify the cost-benefits of these two treatments.

We also have to acknowledge that due to the retrospective nature of the study, we did not retrieve information on adherence to concomitant lipid lowering therapies thus not allowing us to make any consideration on the possible effect of the adherence to background lipid lowering therapies on LDL-C control.

Another possible limitation of our results is the duration of follow-up that was markedly different in the two cohorts. This could be easily explained by the fact that LA treatment is available from 1990 and is authorized for use also in children whereas the availability of lomitapide has only been available for less than 10 years with no authorization to use in minors.

Finally, it is possible that the differences in follow-up duration as well as in baseline characteristics between the two cohorts might also have influenced the comparison of the effect of these two treatments on atherosclerotic burden making any observation on MACE only a conjecture. More studies are needed to clarify the benefit of lomitapide on MACE occurrence.

Considering all these limitations, the clinical implications of our analyses should be considered with much caution, especially because these findings are derived from an indirect comparison and might be biased by several confounding factors.

## Conclusion

In summary, we observed in an indirect comparison that lomitapide appears to be more effective than LA in controlling atherogenic lipoproteins in HoFH. In major guidelines, LA in adjunct to statins and ezetimibe remains the first-choice therapy in the treatment of HoFH [1]. However, our results may open the perspective in which lomitapide, in addition to statins and ezetimibe could be considered as first-line treatment in HoFH. Further studies are necessary in order to formulate an updated protocol for the treatment of HoFH that integrates old and new treatments in a rationale and cost-effective algorithm.

## Material and methods

### Study aim, setting and design

To assess LDL-C reduction, target achievement and long-term LDL-C burden in two HoFH cohorts, one treated with LA, one with Lomitapide, both on top of current available pharmacological treatment. Taking advantage of the early availability of lomitapide in Italy versus later availability in France, we designed a survey in which we retrospectively collected clinical and biochemical information of two independent cohorts of patients with HoFH treated with lomitapide in Italy or LA in France.

### Patients’ selection

HoFH patients known to be receiving lomitapide in Italy were considered for this survey. The inclusion criteria were: (1) molecularly or clinically defined HoFH, (2) age > 18 years, (3) treatment with lomitapide for at least 1 month. All identified patients (n = 34) agreed to participate. Of these, 8 patients were receiving LA plus lomitapide at baseline visit. Since in these patients LA therapy was stopped within 1 year from starting lomitapide and the lipid profile did not differ from that of those receiving lomitapide only, they were kept in the present analysis. Conversely, 4 patients with incomplete lipid profiles during follow-up were excluded. Thus, the Lomitapide cohort included 30 HoFH patients.

The French cohort consisted of all molecularly confirmed HoFH patients referred to the Pitié-Salpêtrière University Hospital who had undergone at least one session of LA. These patients were part of the French Registry of Familial Hypercholesterolemia (REFERCHOL). Details about this registry have been previously reported [28]. A total of 29 French HoFH patients were considered for the present analysis (LA cohort).

### Data collection

For the Lomitapide cohort, physicians were asked to retrospectively revise medical records and extract demographic and clinical information. Details of concomitant lipid-lowering therapy, dosages of lomitapide and baseline plasma lipid values as well as those ones at last follow-up visit were also retrieved. No information on side effects, adherence to medications or diet was obtained.

For the LA cohort, the same data were extracted by two Authors (LD and AG) from REFERCHOL and harmonized for comparison with the Italian database.

Genotypes underlying HoFH in both Italian and French cohort were retrieved by medical records and ascertained as previously reported [6, 14, 23, 24].

Baseline lipid values were defined as those at the date of initiation of lomitapide or LA treatment. Conversely, last follow-up data were defined as those at the time of the last clinic visit as of December 2019. The duration of follow-up was calculated as the difference between last and baseline visit.

Finally, data on MACE at baseline and during follow-up were collected. MACE was defined as a composite of angina, acute myocardial infarction, coronary, carotid or peripheral revascularization (as well as hemodynamic stenosis without revascularization) and ischemic stroke, aortic valve replacement and death for cardiovascular disease [25]. They were identified by either self-reported medical history and/or hospital admission documented in the medical record.

### Laboratory measurements

In both cohorts, blood samples were collected early in the morning after overnight fasting. In HoFH patients undergoing LA, pre- and post-treatment samples were collected. Aliquots of plasma were used to determine total cholesterol (TC), high-density lipoprotein cholesterol (HDL-C) and triglycerides (TG) following standard procedures. LDL-C values were calculated by using the Friedewald’s formula. No complete information was available on ApoB, Lp(a), γGT, CPK and CRP, so that these data were not included in the present analysis.

The estimation of changes in plasma lipid during therapy was carried out using the following reference values: (1) *untreated values*, corresponding to the lipid profile at the worst LDL-C measurement available in medical charts while the patient was not receiving any treatment; (2) *lowest lipid profile on conventional therapies*, estimated as the lipid profile corresponding to the lower LDL-C value with “conventional treatment” (as statins ± ezetimibe ± fibrates ± resins ± porto-caval shunt ± PCSK9i) before the beginning of lomitapide/LA; (3) *baseline values,* corresponding to the time of beginning lomitapide or LA; (4) *last visit values,* corresponding to the last visit when patients were receiving lomitapide or LA up to December 2019. It is worth mentioning that only two patients had weekly LA whereas the remaining were treated on a bi-weekly basis. The overall adherence to LA was on average > 80%, as previously reported [20].

For patients on LA pre- and post-apheresis measurements have been collected to estimate the TC and LDL-C interval means according to the following formula: $Interval mean=[({\mathrm{C}}_{post-LA} + 0.73 \left({\mathrm{C}}_{\mathrm{pre}-\mathrm{LA}}-{C}_{\mathrm{post}-\mathrm{LA}}\right)]$ [26].

The on-treatment cholesterol (${\mathrm{C}}_{\mathrm{follow}-\mathrm{up}}$) was represented by the average of all LDL-C measurements obtained during follow-up in the Lomitapide cohort or by LDL-C interval means in the LA cohort.

### Cholesterol burden estimation

Cholesterol burden was calculated according to the following formula:

$$Total Cholesterol Burden= \sum \begin{array}{c}Cholesterol burden pre-treatment\\ Cholesterol burden on-treatment\end{array}$$

where $$\mathrm{Cholesterol Burden pre}-\mathrm{treatment}= \left({\mathrm{C}}_{\mathrm{baseline}}*{\mathrm{ Age }}_{\mathrm{first LA or Lomitapide prescrption}}\right)$$ $$\mathrm{Cholesterol Burden pre}-\mathrm{treatment}= \left({\mathrm{C}}_{\mathrm{baseline}}*{\mathrm{ Age }}_{\mathrm{first LA or Lomitapide prescrption}}\right)$$ and $$\mathrm{Cholesterol Burden on}-\mathrm{treatment}=({\mathrm{C}}_{\mathrm{follow}-\mathrm{up}}*{\mathrm{Years}}_{\mathrm{follow}-\mathrm{up}})$$ $$\mathrm{Cholesterol Burden on}-\mathrm{treatment}=({\mathrm{C}}_{\mathrm{follow}-\mathrm{up}}*{\mathrm{Years}}_{\mathrm{follow}-\mathrm{up}})$$.

To estimate the average of TC and LDL-C, lipid profiles after baseline were collected every 3 months for the first 3 years, every 6 months for the period between 4 and 10 years and once per year from year 11 until last follow-up (these measurements were available only for patients on LA).

The percentage differences in cholesterol burden were estimated as follows:$$[\frac{cholesterol burden pre-cholesterol burden post}{cholesterol burden pre}] *100$$.

### Statistical analysis

For descriptive statistics, continuous traits were presented as mean and standard deviation or as median and interquartile range as appropriate. Categorical traits were shown as number and proportion. Comparisons were carried out by Mann–Whitney for not-normally distributed and Student’s t-test for normally distributed variables. For differences between categorical traits, *P*-value was calculated by chi-square. Paired T test was used to evaluate the difference between untreated, lowest and last visit total and LDL-C as well as LDL-C burden pre- versus on-treatment.

Linear regression with stepwise method was used to evaluate the association between the variables and adjustments were performed for the following variables: genotype (Null/null mutation versus other), ethnicity (European-non-Finnish vs. other), gender, untreated LDL-C and age at baseline. Not-normally distributed values were *log*-transformed before entering the model.

For the analysis on cardiovascular outcomes, we included only patients that had been exposed to lomitapide or LA for more than 1 year [27]. The number of MACE was counted in each cohort and their incidence rates (IRs) were expressed as number of events per 1,000 patient-year [25]. Cox proportional hazards model was applied to investigate the predictors of incident MACE [25].

Statistical analyses were performed using the IBM Statistical Package for Social Sciences (IBM SPSS, version 25.0, Inc. Chicago, IL). A *P*-value < 0.05 was considered statistically significant.

## Supplementary Information

**Additional file 1: Table 1.** Patients’ genotypes. All mutations were classified according to ACMG guidelines (Chora JR, Medeiros AM, Alves AC, Bourbon M. Analysis of publicly available LDLR, APOB, and PCSK9 variants associated with familial hypercholesterolemia: application of ACMG guidelines and implications for familial hypercholesterolemia diagnosis. Genet Med. 2018;20(6):591-598). For 3 Homozygous LDLR and 1 LDLRAP1 causing mutations were not available and the diagnosis was only on clinical base. *Double Heterozygote patient for mutations in both LDLR (c.373C>T) and PCSK9 (c.60_ 65dupGCTGCT) genes.

**Additional file 2: Figure 1.** LDL-C burden according to lomitapide or LA treatment. A Box plot graphs represent the median values of cumulative LDL-C burden in the Lomitapide cohort (dark grey) and in the LA cohort (light grey). For the total LDL-C burden calculation see Methods. P values are adjusted for age at follow-up, untreated LDL-C values and gender. B, C Box plot graphs represent the median values of TC and LDL-C burden at baseline and on-treatment. For baseline and on-treatment TC or LDL-C burden calculation see Methods. Δ% represents TC and LDL-c percent reduction from baseline and is reported with the respective statistical significance. B shows data form Lomitapide cohort whereas C those from LA cohort.LDL-C, low density lipoprotein cholesterol, LA, Lipoprotein apheresis.

## Data Availability

The datasets used and/or analysed during the current study are available from the corresponding author on reasonable request.

## Abbreviations

ACE: Angiotensin converting enzyme
AE: Adverse events
Apo B: Apolipoprotein B
ASCVD: Atherosclerotic cardiovascular disease
CHD: Coronary heart disease
CPK: Creatin phosphokinase
CRP: C reactive protein
γGT: Gamma glutamil transferase
HDL-C: High-density lipoprotein cholesterol
HoFH: Homozygous familial hypercholesterolemia
IR: Incidence rate
LA: Lipoprotein apheresis
LDL-C: Low-density lipoprotein cholesterol
LDLR: Low-density lipoprotein receptor
LDLRAP1: Low-density lipoprotein receptor associated protein -1
LLT: Lipid lowering treatment
Lp(a): Lipoprotein (a)
MACE: Major atherosclerotic cardiovascular events
MTP: Microsomal triglycerides transferase protein
PAD: Peripheral artery disease
PCSK9i: Proprotein convertase subtilisin/kexin type 9 inhibitors
REFERCHOL: French REgistry of Familial hypERCHOLesterolemia
TC: Total cholesterol
TG: Triglycerides
ULN: Upper limit normal
VLDL: Very-low density lipoproteins
