# Supplementary material for: Long-term efficacy of lipoprotein apheresis and lomitapide in the treatment of homozygous familial hypercholesterolemia (HoFH): a cross-national retrospective survey
Source: Orphanet J Rare Dis. 2021 Sep 8;16:381. doi: 10.1186/s13023-021-01999-8 (PMC8427960; doi:10.1186/s13023-021-01999-8)
Supplement: Supplementary file 1 — Additional file 1: Table 1. Patients’ genotypes. All mutations were classified according to ACMG guidelines (Chora JR, Medeiros AM, Alves AC, Bourbon M. Analysis of publicly available LDLR, APOB, and PCSK9 variants associated with familial hypercholesterolemia: application of ACMG guidelines and implications for familial hypercholesterolemia diagnosis. Genet Med. 2018;20(6):591-598). For 3 Homozygous LDLR and 1 LDLRAP1 causing mutations were not available and the diagnosis was only on clinical base. *Double Heterozygote patient for mutations in both LDLR (c.373C>T) and PCSK9 (c.60_ 65dupGCTGCT) genes. [file 13023_2021_1999_MOESM1_ESM.docx]

**Supplementary Table 1. Patient’s Genotypes**

| **Exon/Intron** | **cDNA** | **Protein change** | **Type of mutation** | **Number of patients** | **Classification by**  **ACMG** |  |
| --- | --- | --- | --- | --- | --- | --- |
| **Homozygous FH causing mutations** | | | | | | |
| exon -1 | c.-156C>T | p.? | promoter | 1 | VUS |  |
| exon -1 | c?-1706_1845+?dup | p.? | duplication | 1 | Pathogenic |  |
| exon 2 | c.97C>T | p.Q33* (Q12X) | stop | 2 | Pathogenic |  |
| intron 1i-12i | c.67+1_68-1_(1845+1_1846-1) | p.(V23Gfs*29) | gross del | 2 | Pathogenic |  |
| exon 3 | c.249-250del | p. P84Sfs*45 | frameshift | 2 | Pathogenic |  |
| exon 4 | c.672C>A | p.D224E | missense | 1 | Likely Pathogenic |  |
| exon 4-9 | - | - | gross deletion | 1 | Pathogenic |  |
| exon 6 | c.828C>G | p.C276W (C255W) | missense | 1 | Likely Pathogenic |  |
| exon 6 | c.859G>T | p.G287C (G266C) | missense | 1 | Likely Pathogenic |  |
| exon 7 | c.1019_1020delinsTG | p.C340L (C319L) | indel | 2 | Pathogenic |  |
| exon 8 | c.1109A>C | p.N370T (N349T) | missense | 1 | Likely Pathogenic |  |
| exon 8 | c.1135T>C | p.C379R (C358R) | missense | 1 | Pathogenic |  |
| exon 9 | c.1291G>A | p.A431T (A410T) | missense | 1 | Pathogenic |  |
| exon 10 | c.1477_1479delinsAGAGACA | p.S493Rfs*44 (S472RfsX44) | frameshift | 1 | Pathogenic |  |
| exon 10 | c.1567G>A | p.V523M (V502M) | missense | 2 | Pathogenic |  |
| exon 11 | c.1618G>A | p.A540T (A519T) | missense | 3 | Likely Pathogenic |  |
| exon 11 | c.1646G>A | p.G549D (G528D) | missense | 1 | Pathogenic |  |
| exon 12 | c.1775G>A | p.G592E (G571E) | missense | 2 | Pathogenic |  |
| exon 14 | c.2043C>A | p.C681* (C660X) | stop | 3 | Pathogenic |  |
| intron 15i | c.2311+1G>A | p.? | splicing | 1 | Pathogenic |  |
| exon 17 | c.2446A>T | p.K816* (K795*) | stop | 1 | Pathogenic |  |
| **Double and Compound Heterozygous causing FH mutations** | | | | | | |
| exon -1 | c.-188C>T | p.? | promoter | 1 | VUS |  |
| exon 4 | c.642G>A | p. W214* (W193X) | stop |  | Pathogenic |  |
| exon 3 | c.259T>G | p. W87G (W66G) | missense | 1 | Likely Pathogenic |  |
| exon 13 | c.1975A>C | p.T659P (T638P) | missense |  | Likely Pathogenic |  |
| exon 3 | c.304C>T | p.Q102* (Q81X) | stop | 1 | Pathogenic |  |
| exon 5 | c.718G>A | p.E240K (E219K) | missense |  | Likely Pathogenic |  |
| exon 3 | c.265T>C | p.C89R (C68R) | missense | 1 | Likely Pathogenic |  |
| exon 12 | c.1775G>A | p.G592E (G571E) | missense |  | Pathogenic |  |
| exon 4 | c.373C>T | p.Q125* (Q104X) | stop | 1 | Pathogenic |  |
| exon 1 | c.60_ 65dupGCTGCT* | p.L22_L23dup | indel |  | VUS |  |
| exon 4 | c.530C>T | p.S177L (S156L) | missense | 1 | Pathogenic |  |
| exon 8 | c.1069 G>A | p.E357K (E336K) | missense |  | Likely Pathogenic |  |
| exon 4 | c.681C>G | p.D227E (D206E) | missense | 1 | Pathogenic |  |
| exon 6 | c.829G>A | p. E277K (E256K) | missense |  | Likely Pathogenic |  |
| exon 9 | c.1268T>C | p.I423T (I402T) | missense |  | Likely Pathogenic |  |
| exon 4 | c.682G>A | p.E228L (E207K) | missense | 1 | Pathogenic |  |
| exon 13 | c.1907G>A | p.G615D (G615D) | missense |  | Likely Pathogenic |  |
| exon 6 | c.858C>A | p.S286 (S265R) | missense | 1 | Likely Pathogenic |  |
| exon 14 | c.2054C>T | p.P685L (P664L) | missense |  | Pathogenic |  |
| exon 6 | c.910G>A | p. D304N (D283N) | missense | 2 | Pathogenic |  |
| exon 11 | c.1681C>T | p.Q561* (Q540X) | stop |  | Pathogenic |  |
| exon 6 | c.910G>T | p.D304Y (D283Y) | missense | 1 | Likely Pathogenic |  |
| exon 11 | c.1633G>A | p.G545R (G524R) | missense |  | Likely Pathogenic |  |
| exon 7 | c.974G>A | p.C325Y (C304Y) | missense | 1 | Likely Pathogenic |  |
| exon 7-15 | - | - | duplication |  | Pathogenic |  |
| exon 8 | c.1135T>C | p.C379R (C358R) | missense | 1 | Pathogenic |  |
| exon 9 | c.1195G>A | p.A399T (A378T) | missense |  | Likely Pathogenic |  |
| exon 9 | c.1201C>G | p.L401V (L380V) | missense | 1 | Likely Pathogenic |  |
| exon 11 | c.1705 G>T | p.D569Y (D548Y) | missense |  | Likely Pathogenic |  |
| exon 11 | c.1618G>A | p.A540T (A519T) | missense | 1 | Likely Pathogenic |  |
| exon 12 | c.1775G>A | p.G592E(G571E) | missense |  | Pathogenic |  |
| exon 11 | c.1646G>A | p.G549D (G528D) | missense | 1 | Pathogenic |  |
| exon 12 | c.1739C>T | p.S580F | missense |  | Likely Pathogenic |  |
| exon 11 | c.1646G>A | p.G549D (G528D) | missense | 1 | Pathogenic |  |
| exon 13-15 | - | - | gross deletion |  | Pathogenic |  |
| exon 12 | c.1775G>A | p.G592E (G571E) | missense | 1 | Pathogenic |  |
| exon 14 | c.2054 C>T | p.P685L (P664L) | missense |  | Pathogenic |  |
| exon 16 | c.2389G>A | p.V797M (V776M) | missense | 1 | Pathogenic |  |
| exon 17 | c.2441G>A | p.R814Q (R793Q) | missense |  | Likely Pathogenic |  |
| **LDLRAP1 mutations causing ARH** | | | | | | |
| exon 4 | c.430_431insA | p.His144fs | 1bp insertion | 5 | Pathogenic |  |
| exon 6 | c.604_605delinsC>A | p.S202H | indel | 1 | Pathogenic |  |
| intron 2i | c.89-1G>C | p.K30Tfs*3 | splicing | 1 | Pathogenic |  |
| exon 4 | c.482_502Dup | p.Cys167_Asp168Ins7 | duplication | 1 | Pathogenic |  |

All mutations were classified according to ACMG guidelines (Chora JR, Medeiros AM, Alves AC, Bourbon M. Analysis of publicly available LDLR, APOB, and PCSK9 variants associated with familial hypercholesterolemia: application of ACMG guidelines and implications for familial hypercholesterolemia diagnosis. Genet Med. 2018;20(6):591-598). For 3 Homozygous LDLR and 1 LDLRAP1 causing mutations were not available and the diagnosis was only on clinical base.

*Double Heterozygote patient for mutations in both LDLR (c.373C>T) and PCSK9 (c.60_ 65dupGCTGCT) genes.
