# Supplementary figures and images for: Long-term efficacy of lipoprotein apheresis and lomitapide in the treatment of homozygous familial hypercholesterolemia (HoFH): a cross-national retrospective survey
Source: Orphanet J Rare Dis. 2021 Sep 8;16:381. doi: 10.1186/s13023-021-01999-8 (PMC8427960; doi:10.1186/s13023-021-01999-8)

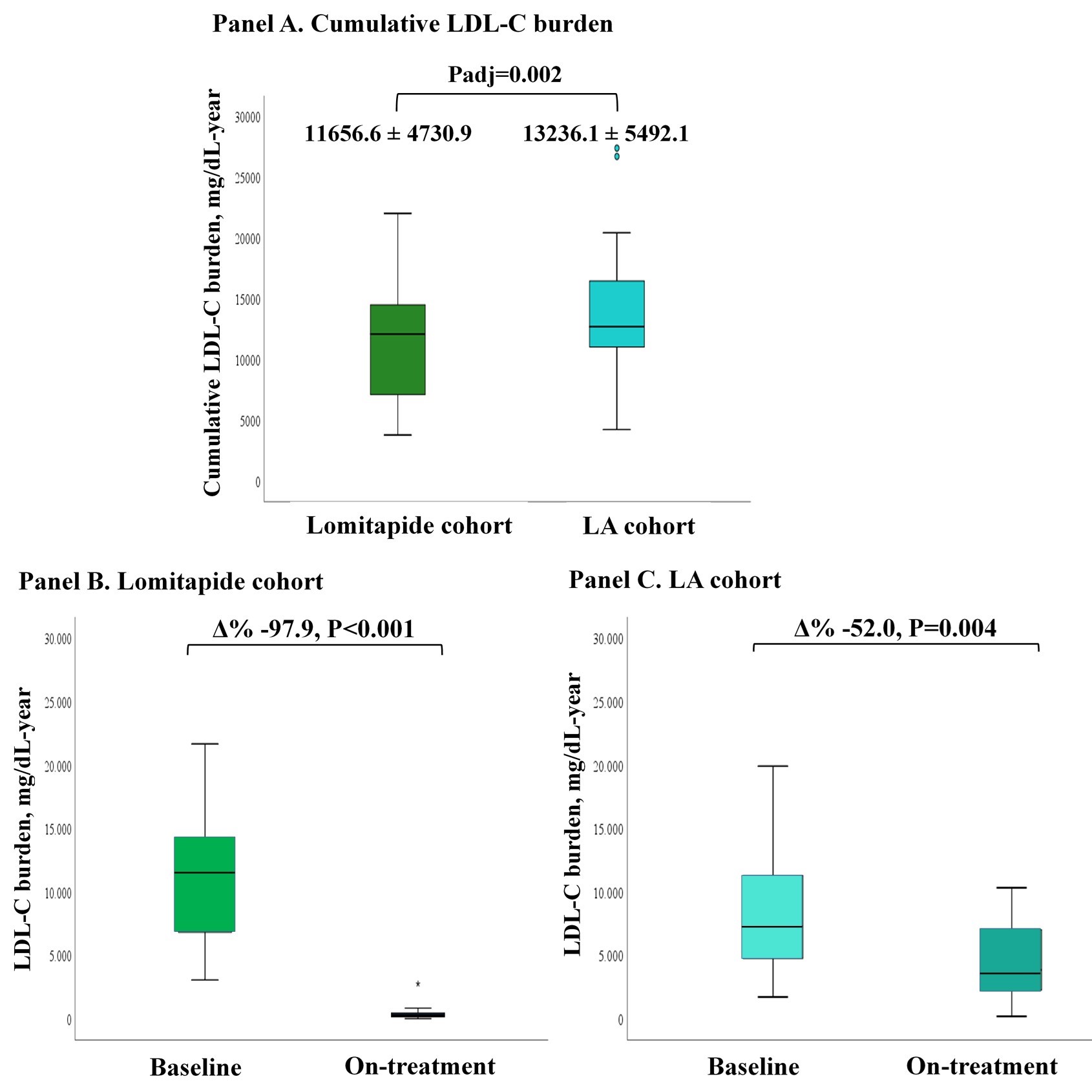

Supplement: Supplementary file 2 — Additional file 2: Figure 1. LDL-C burden according to lomitapide or LA treatment. A Box plot graphs represent the median values of cumulative LDL-C burden in the Lomitapide cohort (dark grey) and in the LA cohort (light grey). For the total LDL-C burden calculation see Methods. P values are adjusted for age at follow-up, untreated LDL-C values and gender. B, C Box plot graphs represent the median values of TC and LDL-C burden at baseline and on-treatment. For baseline and on-treatment TC or LDL-C burden calculation see Methods. Δ% represents TC and LDL-c percent reduction from baseline and is reported with the respective statistical significance. B shows data form Lomitapide cohort whereas C those from LA cohort.LDL-C, low density lipoprotein cholesterol, LA, Lipoprotein apheresis. [file 13023_2021_1999_MOESM2_ESM.jpg]
